# Reciprocal regulation of the *Il9* locus by counteracting activities of transcription factors IRF1 and IRF4

**DOI:** 10.1038/ncomms15366

**Published:** 2017-05-12

**Authors:** Lucia Campos Carrascosa, Matthias Klein, Yohko Kitagawa, Christina Lückel, Federico Marini, Anika König, Anna Guralnik, Hartmann Raifer, Stefanie Hagner-Benes, Diana Rädler, Andreas Böck, Cholho Kang, Michael Lohoff, Holger Garn, Bianca Schaub, Friederike Berberich-Siebelt, Shimon Sakaguchi, Tobias Bopp, Magdalena Huber

**Affiliations:** 1 Institute for Medical Microbiology and Hospital Hygiene, University of Marburg, Marburg 35043, Germany; 2 Institute for Immunology, University Medical Center Mainz, Mainz 55131, Germany; 3 Laboratory of Experimental Immunology, Immunology Frontier Research Center, Osaka University, Osaka 565-0871, Japan; 4 Institute of Medical Biostatistics, Epidemiology and Informatics (IMBEI), University Medical Center Mainz, Mainz 55131, Germany; 5 Institute of Pathology, University of Würzburg, Würzburg 97080, Germany; 6 Institute for Laboratory Medicine and Pathobiochemistry, University of Marburg, Marburg 35043, Germany; 7 Department of Pulmonary and Allergy, University Childrens's Hospital Munich, LMU Munich, Munich 80337, Germany

## Abstract

The T helper 9 (Th9) cell transcriptional network is formed by an equilibrium of signals induced by cytokines and antigen presentation. Here we show that, within this network, two interferon regulatory factors (IRF), IRF1 and IRF4, display opposing effects on Th9 differentiation. IRF4 dose-dependently promotes, whereas IRF1 inhibits, IL-9 production. Likewise, IRF1 inhibits IL-9 production by human Th9 cells. IRF1 counteracts IRF4-driven *Il9* promoter activity, and IRF1 and IRF4 have opposing function on activating histone modifications, thus modulating RNA polymerase II recruitment. IRF1 occupancy correlates with decreased IRF4 abundance, suggesting an IRF1-IRF4-binding competition at the *Il9* locus. Furthermore, IRF1 shapes Th9 cells with an interferon/Th1 gene signature. Consistently, IRF1 restricts the IL-9-dependent pathogenicity of Th9 cells in a mouse model of allergic asthma. Thus our study reveals that the molecular ratio between IRF4 and IRF1 balances Th9 fate, thus providing new possibilities for manipulation of Th9 differentiation.

## Abstract (web summary)

IFN-γ signalling inhibits production of IL-9, the defining cytokine of the Th9 cell subset. Here the authors show that IFN-γ does this by driving IRF1 to compete with IRF4 for *Il9* promoter binding and skewing these cells towards a Th1 phenotype, an effect that reduces asthmatic inflammation in mice.

The generation of T helper (Th) subsets enables specific targeting of pathogens. Signals triggered by antigen recognition, costimulation and cytokines lead to the activation and differentiation of naive T cells by inducing a network of interacting transcription factors that guide their differentiation into distinct Th subsets. The expression of hallmark cytokines characterizes each subset and outlines their specific effector properties^1^. Interferon (IFN)-γ-producing Th1 cells express the master regulator T-bet and promote clearance of intracellular pathogens, whereas Th2 cells secreting interleukin (IL)-4, IL-5 and IL-13 are characterized by the master transcription factor GATA3 and contribute to immunity against helminths. IL-17-, IL-21- and IL-22-producing Th17 cells depend on the lineage-specific transcription factor retinoic acid–related orphan receptor-γt (RORγt) and have a fundamental function in protection from extracellular bacterial and fungal infections. However, Th cell subsets can exert both beneficial and detrimental effects; Th1 and Th17 cells have been implicated in autoimmune tissue inflammation, and Th2 cells can contribute to allergy and asthma^1^,^2^,^3^,^4^,^5^. Furthermore, although Th9 cells (characterized by IL-9 production) are involved in immunity against helminths^6^ and antitumour responses^7^,^8^,^9^, these cells also contribute to immunopathologies, including asthma^10^,^11^,^12^, atopic dermatitis^13^, autoimmunity^14^ and colitis^15^. Hence, unraveling the transcriptional network that regulates Th9 differentiation is pivotal for understanding protective as well as pathogenic effects in atopic and autoimmune diseases.

Th9 cell differentiation is dictated by the cytokine transforming growth factor-β (TGF-β) in combination with IL-4 (refs 6, 16), cytokines that shape the transcriptional Th9 network in concert with T-cell receptor (TCR)-induced and IL-2-induced signals. TGF-β-induced PU.1 binds directly to the *Il9* promoter and probably enhances IL-9 production by modulating permissive histone acetylation at the *Il9* locus^10^,^17^. CD4^+^ T cells deficient in IL-2 do not produce IL-9 and this defect can be reversed by the addition of exogenous IL-2, which induces signal transducer and activator of transcription factor 5 (STAT5)-mediated activation of the *Il9* promoter^18^,^19^,^20^. IL-4 via STAT6 signalling positively regulates Th9 differentiation by enhancing *Il9* promoter activity^21^,^22^ and by upregulating the transcription factor GATA3, which promotes Th9 fate^16^,^23^. Furthermore STAT6 signalling counteracts the IL-9-suppressing transcription factor Foxp3 (refs 16, 24, 25). Importantly, IL-2/STAT5 (ref. 26) and IL-4/STAT6 (ref. 22) as well as TCR signalling^27^ promote the expression of interferon regulatory factor 4 (IRF4), which is essential for Th9 differentiation^11^.

The IRF family of transcription factors consists of nine members; each IRF comprises of a well-conserved DNA-binding domain (DBD), but most IRFs also contain an IRF association domain, which is responsible for homologous as well as heterologous interactions^27^. Compared to other members of the IRF family, IRF4 has lower affinity for the consensus binding motif termed interferon-stimulated response elements (ISRE). IRF4 rather binds cooperatively with other transcription factors to composite regulatory elements^28^,^29^. In conjunction with the activator protein 1 (AP-1) family member BATF, IRF4 binds preferentially to AP-1-IRF4 composite element (AICE) motifs^30^,^31^,^32^,^33^, whereas complexes of IRF4 and proteins from the ETS family, including PU.1, interact at ETS-IRF composite element (EICE) motifs^34^,^35^. IRF4 and BATF are crucial factors for Th9 differentiation^12^ and consequently, IRF4- or BATF-deficient mice are resistant to Th9-dependent allergic airway disease^11^,^12^. The importance of IRF4 is further demonstrated in T cells deficient in the tyrosine kinase Itk, which is an important component of TCR-mediated signalling. Altered TCR signalling in these cells leads to IL-9 inhibition due to attenuated IRF4 expression, which can be rescued by IL-2/STAT5-mediated IRF4 induction^26^. Hence, IRF4 has not only a fundamental role in the differentiation of Th9 but is also known to control Th2, Th17, T follicular helper and T regulatory cell specification^27^.

The first member of the IRF family, IRF1, favours Th1 differentiation in an intrinsic manner by enhancing the expression of IL-12 receptor β1 subunit^36^ and also in a T-cell-extrinsic manner by increasing IL-12 production by antigen-presenting cells^37^,^38^. Conversely, IRF1 suppresses the production of IL-4, IL-5 and IL-13 in Th2 cells^39^,^40^. Thus IRF1 balances Th1 versus Th2 differentiation, and dysregulated *IRF1* consistently associates with atopy^41^ and allergic asthma^42^, immunopathologies linked to both Th2 and Th9 cells. IRF1 is expressed at low levels by most cell types and is induced by various stimuli, including IL-1, lipoploysaccharide, tumour necrosis factor and IFNs. In Th2 cells, IFN-γ stimulation in combination with TCR signalling, strongly upregulates IRF1 expression leading to its increased binding to ISRE, thus inhibiting IL-4 production^39^. Likewise, IFN-γ is known to inhibit IL-9 production via STAT1 (refs 19, 43); however, the exact cellular mediators downstream of STAT1 are not known^44^. Three conserved non-coding sequences (CNS) have been described for the *Il9* locus, CNS0, 1 and 2, whereby the promoter (CNS1) harbours important transcription factor-binding sites for *Il9* regulation^45^. Among them, several ISRE motifs can be found and IRF1 as well as IRF4 bind to the *Il9* promoter^9^,^11^. Considering these findings and that both IRF1 and IRF4 can be upregulated in Th9 cells^9^,^11^, we speculated on a yet unknown interference between these two IRFs during Th9 differentiation.

Here we provide evidence that IFN-γ-mediated upregulation of IRF1 limits IL-9 production in human and mouse Th9 cells. IRF1 suppresses IRF4-driven IL-9 production and counteracts IRF4-driven *Il9* promoter transactivation. Decreased IRF4 abundance correlates with IRF1 binding at the *Il9* locus, suggesting possible competitive binding among these factors. Furthermore, IRF4 and IRF1 display opposing activities on the chromatin state, thus possibly contributing to differential RNA polymerase II recruitment to *Il9*. Genome-wide analyses reveal that IRF1 imprints Th9 cells with a Th1/IFN-associated gene signature, while suppressing Th9 genes. Consistently, IRF1 suppresses Th9 cell-mediated allergic airway disease in an IL-9-dependent manner. Collectively, our data reveal direct opposing activities between IRF members on Th9 differentiation.

## Results

### IRF1 suppresses IRF4-dependent IL-9 production in Th9 cells

**Figure 1:**
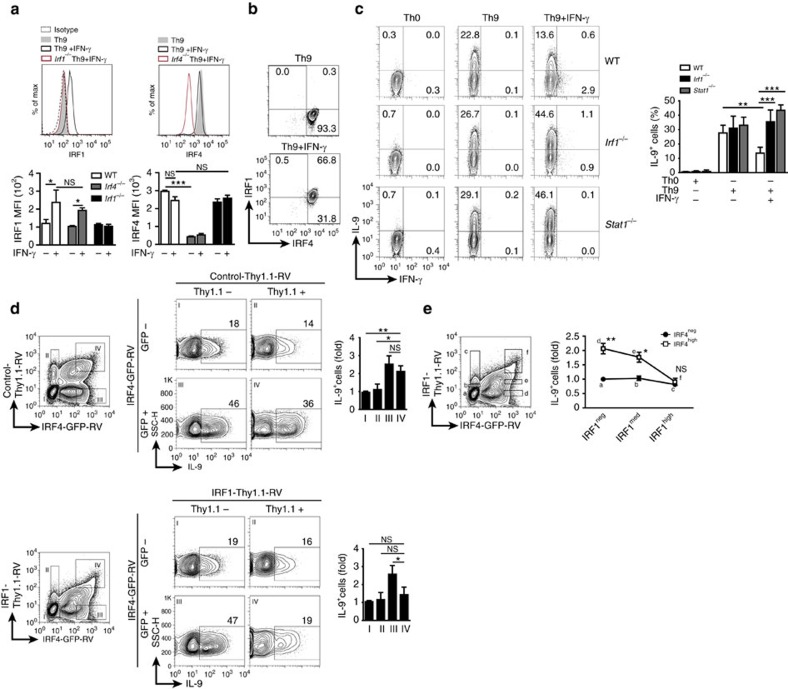
IRF1 limits IRF4-driven IL-9 production dose-dependently. (**a**–**c**) Naive CD44^*−*^CD62L^+^CD4^+^ T cells were isolated from WT, *Irf4*^*−/−*^, *Irf1*^*−/−*^ or *Stat1*^*−/−*^ mice and then treated under Th9 (TGF-β and IL-4) or Th0 (without skewing cytokines) conditions with/without IFN-γ as indicated. (**a**,**b**) After resting and reculture under Th9 conditions for 2 days, intracellular flow cytometric analysis for IRF1 and IRF4 were performed. Bars give geometric mean of fluorescence intensity (MFI). (**c**) Cells were restimulated and then IL-9 and IFN-γ production was detected by flow cytometry, bars to the right give mean of IL-9^+^ T cells. (**d**,**e**) *Irf4*^*−/−*^ CD4^+^ T cells were isolated, activated under Th0 condition overnight and then spin-infected with retroviruses as indicated: IRF4-GFP-RV, control-Thy1.1-RV, and IRF1-Thy1.1-RV. Thereafter, cells were cultured under Th9 conditions for 2 days, rested for 3 days and recultured under Th9 conditions for additional 2 days. Four subsets (I–IV) were selected for analysis of IL-9 production. Bars to the right show fold induction of IL-9^+^ T cells relative to GFP^*−*^Thy.1.1^*−*^ cells (subset I). (**e**) Six subsets (a through f) expressing increasing levels of GFP and Thy1.1 were selected for analysis of IL-9 production (left panel). Dot plot to the left is representative for four independent experiments. Graph to the right shows fold induction of IL-9^+^ T cells relative to GFP^*−*^Thy1.1^*−*^ cells (subset a) combined from four independent experiments. Histogram and contour-plots are representative of two (**a**,**b**), three (**c**) or four (**d**,**e**) independent experiments. Bars show mean±s.d. from combined two (**a**,**b**), three (**c**) or four (**d**,**e**) experiments. *P<0.05, **P<0.005, ***P<0.001 (two-tailed Student's *t*-test).

To examine whether there is a mutual regulation between IRF1 and IRF4 expression in Th9 cells, we determined IRF1 and IRF4 protein levels in the presence or absence of IFN-γ, which is a known IRF1 inducer^46^. As expected, IRF1 was strongly upregulated by IFN-γ in Th9 cells (Fig. 1a,b) in a STAT1-dependent manner (Supplementary Fig. 1a,b). The upregulation of IRF1 by IFN-γ did not affect the levels of co-expressed IRF4 and vice versa (Fig. 1a,b). Furthermore, neither the expression of several transcription factors involved in Th9 differentiation^10^,^12^,^16^,^18^,^21^,^22^,^23^,^24^,^25^ including PU.1 (encoded by *Spi1*), BATF, GATA-3 and T-bet nor the phosphorylation levels of STAT3 and STAT5 were significantly affected by IRF1-deficiency (Supplementary Fig. 1c–g). The expression of Foxp3, which represses Th9 differentiation^16^,^22^,^25^, was slightly upregulated in *Irf1*^*−/−*^ as compared to wild-type (WT) Th9 cells (Supplementary Fig. 1h).

Although IRF1 did not interfere with the levels of IRF4 and other transcription factors contributing to Th9 differentiation, it was still conceivable that it affects IL-9 expression in Th9 cells by other mechanisms. To test this possibility, we sorted naive CD62L^+^CD44^*−*^CD4^+^ WT, *Irf1*^*−/−*^ and *Stat1*^*−/−*^ cells, cultured them under Th9-skewing conditions in the presence or absence of IFN-γ and analysed IL-9 production. IFN-γ suppressed the proportion of IL-9-producing WT cells, which is consistent with previous findings^19^, while it rather supported the generation of IL-9-producing cells in the absence of IRF1 or STAT1 (Fig. 1c), indicating that IFN-γ suppressed IL-9 production in Th9 cells via STAT1-induced IRF1. Thus, in contrast to the known positive regulation of IL-9 by IRF4^2^, IRF1 displayed an opposite effect when induced by IFN-γ, implying that there might be an interference between these two transcription factors during IL-9 regulation in Th9 cells.

To proof this hypothesis, we co-transfected *Irf4*^*−/−*^ Th9 cells, which produce low amounts of IL-9, with a retroviral vector encoding IRF4 tagged with green fluorescent protein (GFP) (IRF4-GFP-RV) and with a vector encoding Thy1.1-tagged IRF1 (IRF1-Thy1.1-RV) or with corresponding control empty vectors (Control-Thy1.1-RV, Control-GFP-RV). Then the cells were analysed for IL-9, IRF1 and IRF4 expression. While cells that were transduced with neither virus displayed low IL-9 positivity (Thy1.1^*−*^GFP^*−*^), as expected, a substantial proportion of the cells transduced with IRF4-GFP-RV (Thy1.1^*−*^GFP^+^) highly produced IL-9 (Fig. 1d). This effect was reversed in cells that had been co-infected with IRF1-Thy1.1-RV (Thy1.1^+^GFP^+^, Fig. 1d, bottom panel). The suppression was specific for IRF1 because the Control-Thy1.1-RV did not diminish IRF4-driven IL-9 production significantly (Thy1.1^+^GFP^+^, Fig. 1d, upper panel). Importantly, IRF1-mediated inhibition of IL-9 production was dose-dependent and restricted to IRF4-promoted IL-9 production (Fig. 1e). The high expression of GFP and Thy1.1 correlated with high IRF1 and IRF4 expression in *Irf4*^*−/−*^ Th9 cells transduced with IRF4-GFP-RV and IRF1-Thy1.1-RV, respectively (Supplementary Fig. 2a). Of note, the relative intensities of GFP and Thy1.1 tags representing the relative expression of IRF1 and IRF4 correlated with IRF4 and IRF1 protein levels (Supplementary Fig. 2b,c). Thus these results imply that IRF1 suppresses IRF4-driven IL-9 production in a quantitative manner.

### IRF1 and IRF4 control the *Il9* locus

**Figure 2:**
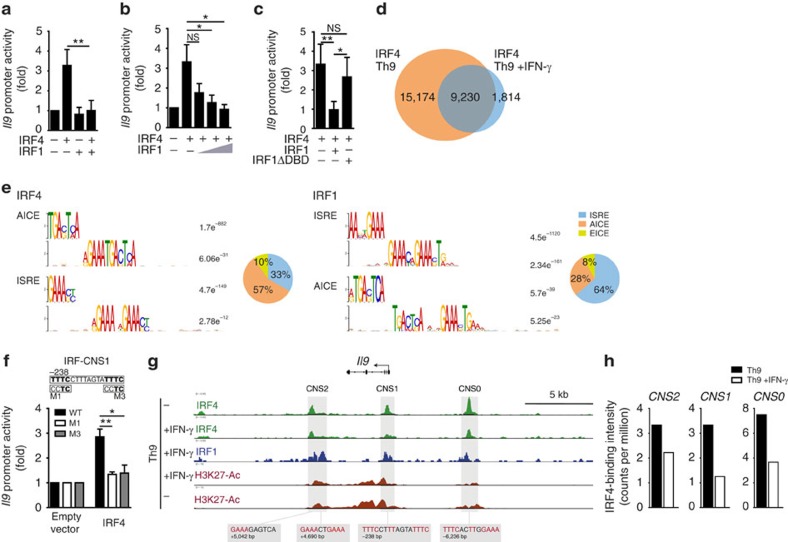
Increased IRF1 binding correlates with decreased IRF4 binding at the *Il9* locus. (**a**–**c**) Luciferase reporter assay of Th0-polarized cells transiently transfected with constant amounts of *Il9* promoter-luciferase vector and with either empty vector (−), IRF4-expressing vector (IRF4) and/or: (**a**) constant amounts of IRF1-expressing vector (IRF1), (**b**) increasing amounts of IRF1 (0, 0.5, 1, 2 μg) or (**c**) IRF1 or an IRF1 mutant lacking the DNA-binding domain (ΔDBD). (**d**,**e**,**g**,**h**) ChIP-Seq analyses of Th9 cells with or without IFN-γ treatment. Cells were crosslinked with formaldehyde and immunoprecipitated with the indicated antibodies. Massive parallel sequencing of immunoprecipitated DNA was performed. (**d**) Numbers in Venn diagram indicate the IRF4-binding peaks. (**e**) IRF4 binding in Th9 cells and to the right IRF1 binding in Th9 cell treated with IFN-γ were analysed. Over-represented motifs shown as PWM (positional weight matrix) revealed by MEME/DREME analysis are shown. Associated piecharts indicate the frequency of designated motifs. (**f**) Non-polarized Th0 cells were transfected with either WT or the mutated M1 or M3 *Il9* promoter-luciferase vectors (substitution mutation within the *Il9* promoter IRF site is depicted above) together with empty vectors and/or IRF4. (**g**) ChIP-Seq analyses show IRF4 (upper lanes), IRF1 (middle lane) or H3K27-Ac (bottom two lanes) abundance. Below in grey boxes, potential IRF-binding sites are depicted including their distance from the *Il9* transcription start site. (**h**) Normalized read counts from IRF4-ChIP-Seq in Th9 cells in the absence or presence of IFN-γ for CNS0, 1 and 2 are shown. (**a**–**c**,**f**) Data are combined from three independent experiments and show mean±s.d. *P<0.05, **P<0.005, ***P<0.001 (two-tailed Student's *t*-test).

Since IRF4 and most transcription factors related to *Il9* locus regulation exert their function through direct binding to the *Il9* promoter^45^, we analysed IRF4 and IRF1 binding and their functional interference at the *Il9* promoter. For this, we performed reporter assays in non-polarized CD4^+^ T cells transfected with an *Il9* promoter-luciferase construct (pGL3-*Il9*) and IRF1- and/or IRF4-encoding plasmids. Co-transfection of the *Il9* promoter-luciferase construct with IRF4 resulted in transactivation (Fig. 2a), as previously described^11^. Consistent with the repressive activity of IRF1 on IRF4-mediated IL-9 production, the co-transfection of IRF1 suppressed IRF4-driven *Il9* promoter activity in non-polarized CD4^+^ T cells (Fig. 2a). The inhibitory IRF1 effect was dose- dependent and relied on the DBD (Fig. 2b,c), suggesting that IRF1 might directly interfere with the transactivating effects of IRF4 at the *Il9* promoter. We further confirmed the role of IRF1 DBD on IL-9 production in Th9 cells using overexpression of retroviral vectors encoding IRF1ΔDBD (IRF1ΔDBD-GFP-RV), IRF1 (IRF1-GFP-RV) or control vector (Control-GFP-RV) (Supplementary Fig. 3a,b).

In order to evaluate the underlying molecular mechanisms of IRF1- and IRF4-dependent *Il9* regulation, we performed chromatin immunoprecipitation (ChIP) with IRF4- and IRF1-specific antibodies in Th9 cells followed by sequencing (ChIP-Seq) as described^47^. To determine the permissive histone marks of regions bound by IRF1 or IRF4, we included ChIP-Seq analyses for acetylated histone H3 at Lys27 (H3K27-Ac), which is found in active regulatory regions^48^. We identified high-confidence genome-wide binding regions for IRF4; interestingly approximately 60% of these IRF4 peaks were lost upon IFN-γ signalling (15,174 of the 24,457 IRF4 peaks lost, Fig. 2d). We analysed the occurrence of AICE, EICE and ISRE motifs coinciding with IRF1 and IRF4 peaks. As previously described^30^,^31^,^32^,^33^, IRF4 binding highly associated with AICE motif prevalence, whereas ISRE as well as EICE motifs were rather bound with lower frequency by IRF4 in Th9 cells (Fig. 2e). In contrast, IRF1 peaks highly correlated with ISRE occurrence in Th9 cells upon IFN-γ treatment, as expected (Fig. 2e). Consistent with motif analyses, IRF1 and IRF4 binding did overlap in some regions (Supplementary Fig. 3c).

Next we focused on the *Il9* locus and determined IRF4-binding sites within CNS1. Considering IRF4 preferential genome-wide binding to AICE, we surprisingly found that two mutations (termed M1 and M3) in ISRE motifs (in the region termed IRF-CNS1) abrogated IRF4-promoted *Il9* promoter activity in luciferase assays indicating that these ISRE are crucial for positive regulation of *Il9* promoter activity by IRF4 (Fig. 2f). Interestingly, we detected higher IRF4-binding density in Th9 cells than in Th9 cells treated with IFN-γ mainly at CNS1 and CNS0 and (to a smaller extent) also at CNS2 of the *Il9* locus (Fig. 2g,h). In contrast, IRF1 binding to the *Il9* locus was induced by treatment of Th9 cells with IFN-γ (Supplementary Fig. 3d). IRF1 bound at CNS1 as previously described^9^ as well as at CNS0 and CNS2 (Fig. 2g). Thus decreased IRF4 abundance correlated with the appearance of IRF1 peaks at the same regions located in the *Il9* locus and this was accompanied by slightly decreased H3K27-Ac occupancy (Fig. 2g). These results suggest that IRF1 might impair IL-9 production by competing with IRF4 binding at ISRE in the *Il9* locus.

### Opposite regulation of histone marks by IRF1 and IRF4

**Figure 3:**
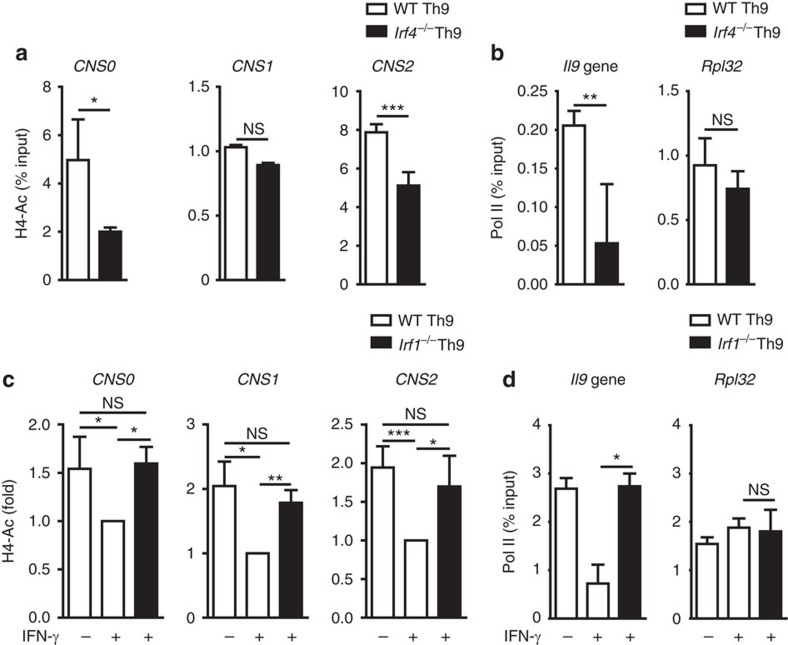
Opposite effects of IRF1 and IRF4 on histone acetylation at *Il9* locus. (**a**–**d**) Purified WT and (**a**,**b**) *Irf4*^*−/−*^ or (**c**,**d**) *Irf1*^*−/−*^ CD4^+^ T cells were cultured under Th9 conditions with or without IFN-γ. (**a**,**c**) ChIP analysis of histone H4 acetylation (Ac) at CNS of the *Il9* gene. (**c**) Bars show fold induction of H4-Ac relative to WT Th9 cells treated with IFN-γ. (**b**,**d**) ChIP analysis for RNA polymerase II (pol II) occupancy at *Il9* and *Rpl32* genes. (**a**–**d**) The same chromatin was used for control ChIP experiments with control IgG. Precipitated DNA is presented relative to input (% input). Values for nonspecific binding (as determined by using control IgG) were subtracted. (**a**–**d**) Data are shown as mean±s.d. of combined results from two independent experiments (triplicates to quintuplets in qRT-PCR). The experiments were repeated (**b**) three or (**d**) five times with consistent results. *P<0.05, **P<0.005, ***P<0.001 (two-tailed Student's *t*-test).

For efficient transcription, the locus must be held in an open conformation, which can be monitored by characteristic histone modifications, for example, acetylation^49^. In Th9 cells, the acetylation of histones H3 and H4 at *Il9* CNS is associated with enhanced *Il9* transcription^10^,^25^. Reciprocal binding of IRF1 and IRF4 to the *Il9* locus accompanied by differential regulation of IL-9 production suggested that these transcription factors might alter epigenetic modifications. ChIP-Seq analysis (Fig. 2g) supported this hypothesis to some extent. To closer approach this issue, we performed ChIP followed by quantitative real time-PCR (qRT-PCR) analysis at *Il9* CNS for total acetylation of histone H4 (H4-Ac). We found less H4-Ac at the CNS0 and CNS2 in *Irf4*^*−/−*^ Th9 cells as compared to WT Th9 cells (Fig. 3a) and this correlated with decreased RNA polymerase II (pol II) abundance at the *Il9* gene (Fig. 3b), suggesting that the positive regulation of the acetylation status by IRF4 supports *Il9* transcription. In contrast, IFN-γ treatment suppressed H4-Ac at *Il9* CNS in WT but not in *Irf1*^*−/−*^ Th9 cells (Fig. 3c, Supplementary Fig. 4a). This was accompanied by a decreased pol II recruitment to the *Il9* gene upon IFN-γ treatment in WT Th9 cells, and this effect was reversed by IRF1 deficiency (Fig. 3d). This implies that negative regulation of histone acetylation state by IRF1 may contribute to the restricted *Il9* transcription. In non-stimulated WT and *Irf1*^*−/−*^ CD4^+^ T cells, no differences in H4-Ac at *Il9* CNS were detectable, indicating that the described differences in the H4-Ac status were not caused by developmental alterations (Supplementary Fig. 4b). To analyse whether IRF1 suppresses histone acetylation by recruitment of histone deacetylases (HDAC), we applied the HDAC inhibitor trichostatin A during Th9 polarization in the presence or absence of IFN-γ. Under both conditions, we observed similar increase in IL-9 production by Th9 cells upon addition of the inhibitor (Supplementary Fig. 4c). This indicates that IRF1 suppresses chromatin accessibility independently of HDAC recruitment to the *Il9* locus. These data reveal an opposite regulation of histone acetylation at *Il9* CNS by IRF4 and IRF1.

### IRF1 imprints Th9 cells with IFN-γ-/Th1-associated signature

**Figure 4:**
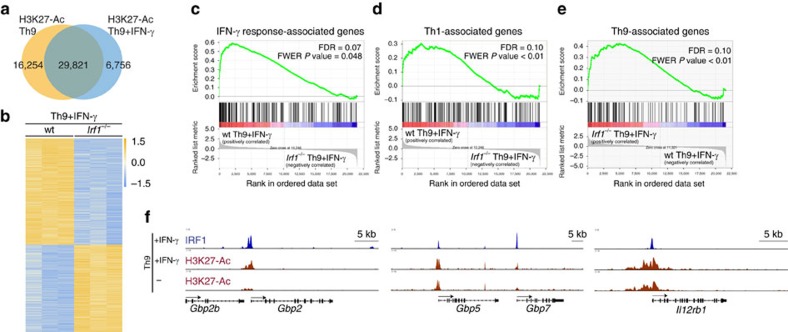
The IFN-γ/IRF1 pathway upregulates IFN/Th1-associated genes in Th9 cells. (**a**) ChIP-Seq analyses of Th9 cells with or without IFN-γ treatment. Cells were crosslinked with formaldehyde and immunoprecipitated with anti-H3K27-Ac antibodies. Massive parallel sequencing of immunoprecipitated DNA was performed. Numbers in the Venn diagram represent H3K27-Ac peaks. (**b**) Naive CD4^+^ T cells isolated from WT and *Irf1*^*−/−*^ mice were cultured under Th9 conditions in the presence of IFN-γ. Total RNA was purified from the cells and RNA-Seq was performed (three independent biological samples). Heatmap shows colour-coded *z*-scored fragments per kilobase of exon per million fragments mapped (*P*<0.01). (**b**–**d**) GSEA was performed according to the Broad institute with (**c**) the hallmark IFN-γ response, (**d**) Th1 upregulated genes and (**e**) Th9 upregulated genes. (**f**) ChIP-Seq analysis of Th9 cells with or without IFN-γ treatment. Cells were crosslinked with formaldehyde and immunoprecipitated with anti-IRF1 (upper lanes) or anti-H3K27Ac antibody (middle and bottom lanes). Transcription start site of genes are indicated below as arrows.

On a genome-wide level, IFN-γ signalling caused both hypoacetylation and hyperacetylation on loci in Th9 cells, indicating a differential regulation of regulatory regions (Fig. 4a). To understand the extent to which IRF1 contributes to transcriptional programming of Th9 cells on a global level, we performed RNA sequencing (RNA-Seq) from WT or *Irf1*^*−/−*^ Th9 cells treated with IFN-γ. There were 735 significantly downregulated, while 604 significantly upregulated transcripts (*P*<0.01) in *Irf1*^*−*/*−*^ relative to WT Th9 cells (Fig. 4b), indicating that IRF1 signalling negatively and positively regulates genes downstream of IFN-γ. Gene-set enrichment analysis (GSEA) using Molecular Signatures Database (MSigDB, Broad Institute) as well as published gene expression signatures^12^ revealed that positively regulated genes by IRF1 signalling (those upregulated in WT as compared to *Irf1*^*−/−*^ Th9 cells) were enriched for IFN-γ response genes and Th1 signature-associated genes (Fig. 4c,d, Supplementary Fig. 5a,b). In contrast, the negatively regulated genes (those downregulated in WT cells as compared to *Irf1*^*−/−*^ Th9 cells) were enriched for genes associated with a Th9 signature (Fig. 4e, Supplementary Fig. 5c). Hence, IFN-γ-IRF1 signalling promotes a transcriptional shift of Th9 cells towards an IFN-γ/Th1 signature. This was further confirmed by functional enrichment analysis on sets of genes derived from IRF1 promoter-binding profiles. A majority of pathways linked to the IFN/Th1 signature overlapped between GSEA and ChIP-Seq-based gene ontology (GO) enrichment analysis of IRF1 targets, including viral response pathways as well as IL-12- and IFN-associated pathways (Supplementary Data 1). Among the genes positively regulated by IRF1 signalling and directly bound by IRF1, we found the already known targets, guanylate-binding protein 2 (*Gbp2*) and *Gbp5* (refs 50, 51), which contribute to clearance of infection as well as Th1-associated genes, including *Il12rb1* (refs 1, 36; Fig. 4f). Collectively, the data suggest that IRF1 can act as a direct activator of IFN/Th1 pathways, implying that IRF1 signalling modulates the transcriptional fate of Th9 cells by enriching IFN-γ/Th1-associated gene signatures, while limiting Th9-associated genes.

### IRF1 limits allergic airway inflammation IL-9 dependently

**Figure 5:**
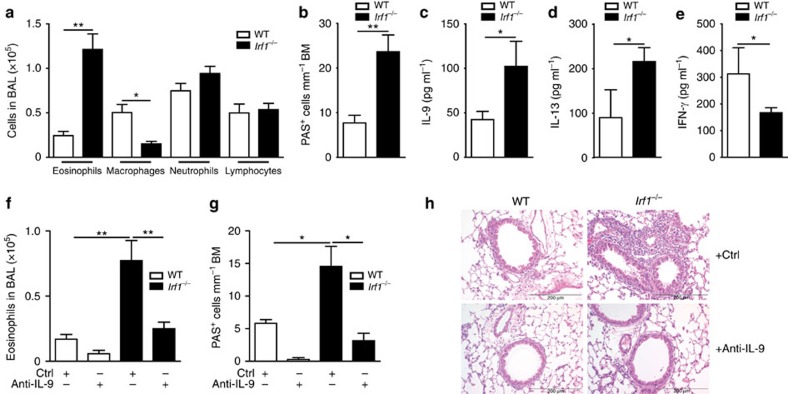
The IFN-γ/IRF1 pathway restricts Th9-dependent allergic airway inflammation. WT or *Irf1*^*−/−*^ Th9 cells treated with IFN-γ were adoptively transferred into *Rag2*^*−/−*^ mice, which were then challenged with nebulized OVA in the absence or presence of IL-9-neutralizing monoclonal antibodies (anti-IL-9) or control rat IgG (Ctrl) antibodies as outlined in Supplementary Fig. 6. (**a**,**b**,**f**,**g**) Cell numbers in the BAL fluid are displayed. (**b**,**g**) Numbers of mucus-producing goblet cells evaluated by periodic acid-Schiff staining. (**c**–**e**) Lung cells were stimulated with 2 mM OVA_323–339_ for 3 days. IL-9, IL-13 and IFN-γ production was determined in supernatants by ELISA. (**h**) Tissue inflammation was evaluated with haematoxylin and eosin staining, original magnification × 100. (**a**–**g**) Data are shown as the mean±s.e.m. of 10–15 mice combining two independent experiments. (**a**–**h**) The experiments were repeated four times with consistent results. *P<0.05, **P<0.005 by one-way ANOVA with Tukeýs post-test (**a**,**b**,**f**,**g**) or one-tailed Student's *t*-test (**c**–**e**).

As *IRF1* single-nucleotide polymorphisms associate with childhood allergic asthma^42^ and IL-9 production is postulated to be an initial event promoting allergy^52^, we analysed the role of IRF1 in a murine model of allergic asthma. However, allergy studies directly in *Irf1*^*−/−*^ mice will not be informative due to defects in antigen-presenting cells^37^,^46^ in addition to T-cell-intrinsic alterations. To circumvent this difficulty, we polarized WT and *Irf1*^*−/−*^ OTII cells, carrying a transgenic TCR specific for chicken ovalbumin (OVA), under Th9 conditions in the presence of IFN-γ. Thereafter, the cells were injected into mice lacking T and B cells (*Rag2*^*−/−*^), which were subsequently challenged with OVA to provoke asthma symptoms (Supplementary Fig. 6a). *Irf1*^*−/−*^ compared to WT OTII Th9 cells caused significantly increased eosinophil numbers in broncho-alveolar lavage (BAL) fluid, while macrophage numbers were decreased (Fig. 5a). This was accompanied by increased presence of mucus-producing cells in recipients of *Irf1*^*−/−*^ compared to WT OTII Th9 cells (Fig. 5b). Additionally, OVA-stimulated lung cells isolated from recipients of *Irf1*^*−/−*^ compared to WT OTII Th9 cells produced more IL-9 and IL-13 but less IFN-γ (Fig. 5c–e), revealing that *Irf1*^*−/−*^ Th9 cells retain their pro-allergic phenotype *in vivo* as compared to WT Th9 cells. The pro-allergic function of *Irf1*^*−/−*^ Th9 cells was ameliorated by treatment with IL-9-neutralizing antibodies as determined by decrease in eosinophilia, in the numbers of mucus-producing cells and in inflammation (Fig. 5f–h, Supplementary Fig. 6b,c). Accordingly, the amounts of secreted IL-9 and IL-13 (which is regulated by IL-9 in asthma^53^) were strongly diminished by IL-9 neutralization (Supplementary Fig. 6d,e). Thus IFN-γ/IRF1 signalling restricts Th9-mediated airway allergy in an IL-9-dependent manner while enhancing IFN-γ production in Th9 cells, indicating that IRF1 constrains allergic airway disease by skewing Th9 cells towards a ‘low-allergic' IFN/Th9 phenotype.

### IRF1 suppresses IL-9 production in human Th9 cells

**Figure 6:**
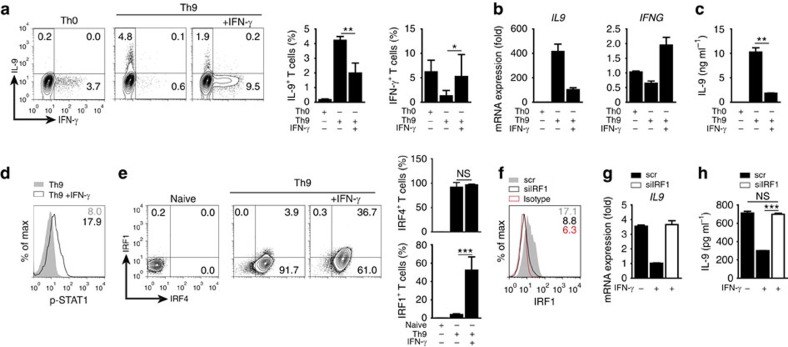
IFN-γ/STAT1-induced IRF1 suppresses IL-9 production in human Th9 cells. Naive CD4^+^CD25^*−*^CD45RA^+^CD45RO^*−*^ T cells were isolated from human PBMCs and cultured under Th0 or Th9 conditions in the presence or absence of human IFN-γ as indicated. (**a**) Intracellular staining for IL-9 and IFN-γ after 3 days of culture (bars to the right give mean±s.d. of three independent experiments). (**b**) Expression of *IL9* and *IFNG* mRNA on day 2 in Th0 and Th9 cells; results were normalized to *18S* and are presented relative to Th0 cells. (**c**) ELISA of IL-9 in supernatants of Th0 and Th9 cultures after 3 days. (**d**) Phospho-flow for p-STAT1 in Th9 cells with or without IFN-γ treatment, day 2 of culture (values show mean fluoresence intensity). (**e**) Intracellular staining for IRF1 and IRF4, day 2 of culture (bars to the right give mean±s.d. of three independent experiments). (**f**–**h**) Human naive CD4^+^CD25^*−*^CD45RA^+^CD45RO^*−*^ T cells were activated under Th0 condition for 16 h, then transfected with scrambled (scr) or IRF1-specific siRNA (siIRF1) and then cultured for further 72 h under Th9 conditions with/without IFN-γ. (**f**) Intracellular staining of IRF1 in scr- or siIRF1-transfected cells 24 h post transfection upon culture under Th9 conditions with IFN-γ. (**g**) Expression of *IL9* mRNA in transfected cells at 48 h post transfection; results were normalized to 18S and are presented relative to Th9 cells treated with scr siRNA and IFN-γ. (**h**) ELISA of IL-9 in supernatants of transfected cells 72 h post transfection. (**a**–**h**) Data are representative of three independent experiments with different donors; *P<0.05, **P<0.005, ***P<0.001 (two-tailed Student's *t*-test).

Finally, we investigated whether IRF1 also regulates IL-9 production in human Th9 cells. For this, we isolated naive CD4^+^CD25^*−*^CD45RA^+^CD45RO^*−*^ T cells from peripheral blood mononuclear cells (PBMCs) of healthy human subjects and activated them in the absence of skewing cytokines (Th0 conditions) or in the presence of TGF-β plus IL-4 (Th9 conditions) with or without addition of IFN-γ. As already reported^11^,^54^, Th9 conditions induced IL-9 production by CD4^+^ T cells, whereas the addition of IFN-γ counteracted this effect and simultaneously upregulated IFN-γ (Fig. 6a). This was confirmed by qRT-PCR and detection of IL-9 in supernatants of cultured cells (Fig. 6b,c). Similar to murine Th9 cells (Supplementary Fig. 1a), the addition of IFN-γ caused increased phosphorylation of STAT1 also in human Th9 cells (Fig. 6d) and this was accompanied by upregulation of IRF1, which was co-expressed with IRF4 (Fig. 6e). Knockdown of *IRF1* expression by siRNA (Fig. 2f) abolished IFN-γ-mediated suppression of *IL9* mRNA expression and IL-9 production in human Th9 cells (Fig. 6g,h). Thus IFN-γ signalling requires IRF1 to suppress IL-9 production in human Th9 cells. This points to the existence of comparable programmes dictated by the IFN-γ-STAT1-IRF1 pathway suppressing IL-9 production in human and murine Th9 cells.

## Discussion

Th9 cells exert protective as well as pathophysiological immune functions^44^; however, the detailed regulation of the Th9 transcriptional network remains incompletely understood. Here we show that IRF1 limited IL-9 production downstream of IFN-γ/STAT1 signalling in mouse and human Th9 cells. IRF1 repressed IRF4-driven IL-9 production and *Il9* promoter activity in a quantitative manner. Direct IRF1 binding at the *Il9* promoter was associated with decreased abundance of IRF4 and reduced acetylation levels as well as limited RNA polymerase recruitment at the *Il9* locus. These results suggest that the suppressive function of IRF1 is probably mediated by a direct binding competition with IRF4 at the *Il9* locus, resulting in opposite regulation of histone acetylation and associated *Il9* transcription. IFN-γ-induced IRF1 evoked a shift in Th9 cells towards an IFN/Th1-like signature through direct and indirect mechanisms. Accordingly, the absence of IRF1 in IFN-γ-treated Th9 cells led to increased IL-9-dependent pathogenicity in AAD, a mouse model for allergic asthma. Thus the transcriptional network of Th9 differentiation is shaped by opposing actions of two IRF family members, IRF1 and IRF4.

Th9 cells contribute to human atopic diseases and accordingly Th9-related genes associate with asthma development^44^. We demonstrated that IFN-γ upregulates IRF1 in human Th9 cells and that IRF1 upregulation correlated with decreased IL-9 production. Furthermore, silencing of IRF1 restored IFN-γ-mediated IL-9 suppression, indicating that IRF1 counteracts IL-9 production also in human Th9 cells. Indeed, *IRF1* single-nucleotide polymorphisms associate with asthma development and atopy^41^,^42^. In addition, increased levels of *IRF4* and IL-9 were accompanied by diminished *IFNG* expression in the peripheral blood of allergic asthmatics^55^. Given those findings, one could speculate that asthmatics might fail to upregulate IRF1 upon infection-induced IFN-γ signalling leading to exacerbated asthma due to defects in Th9 and Th2 inhibition. However, further work must be carried out to fully understand the impact of *IRF1* signalling on Th9 cells during asthma exacerbations.

To induce asthma pathophysiology in the mouse model, we performed adoptive transfer experiments of OVA-transgenic IFN-γ-treated WT and *Irf1*^*−/−*^ Th9 cells into *Rag2*^*−/−*^ mice and subsequently challenged the mice with nebulized OVA. Indeed, the absence of IRF1 resulted in more severe AAD, which was dependent on IL-9 as shown by neutralization with anti-IL-9 antibody. Besides decreasing IL-9 production, we wondered whether IRF1 as a transcription factor linked to Th1 differentiation also affects other functionally relevant genes. Therefore, we made use of genome-wide RNA-Seq to investigate the complex IRF1-mediated transcriptional interplay in IFN-γ-treated Th9 cells. Interestingly, GSEA revealed an IRF1-dependent and IFN-γ-induced shift towards a Th1/IFN-like phenotype, while suppressing Th9 signature genes. Pathways associated with infectious and interferon response^36^,^50^,^51^,^56^, for example, *Gbp* and *Il2rb1* were directly upregulated, indicating that IRF1 balances Th9 versus Th1-like plasticity by its ability to act as a transcriptional repressor as well as an activator. Recently, Végran *et al*.^9^ published that IRF1 is a positive regulator of IL-9 and Th9 differentiation when induced by IL-1β and dictates IL-21-dependent antitumour function in a melanoma model. However, there are many possible explanations for this discrepancy to our study. The cytokine signalling triggered by IL-1β differs greatly from that of IFN-γ, therefore it is conceivable that the cellular milieu contains distinct transcription factors that might interfere with IRF1 functions. Among these, nuclear localization of the IL-9-promoting transcription factor nuclear factor-κB^24^,^25^,^57^ is induced by IL-1β signalling^58^, which might alter IRF1 actions. To exclude the influence of the microbial environment of the animal facilities, we extended our work to human Th9 cells where we demonstrated that IRF1 indeed inhibited IL-9 expression in the context of IFN-γ signalling. We therefore conclude that IFN-γ-IRF1 signalling suppresses the Th9 fate in humans and mice.

IRF family members are known to regulate gene expression positively or negatively in different cell types, including Th cells^46^. To carry out these effects, IRFs can interact via the IRF association domain with family members or with proteins belonging to other families. For example, IRF1-IRF8 heterodimers mostly act as transcriptional repressors at ISRE, whereas IRF1, IRF3 and IRF7 form an IFN-β enhanceosome together with nuclear factor-κB, AP-1 and CBP/p300 to induce *Ifnb* expression^59^. Only few studies describe antagonizing functions of IRFs at ISRE in gene regulatory. It has been described that IRF8 represses IRF9 binding to ISRE at IFN-stimulated gene 15 (ref. 29), while IRF4 counteracted IRF1-driven tumour necrosis factor-related apoptosis-inducing ligand promoter activity in a DBD-dependent manner^60^. Furthermore, IRF1 opposes IRF4-driven *Il17* promoter activity, and conversely, IRF4 antagonizes IRF1-mediated *Il10* promoter activity^61^.

In our study, we propose a model in which IRF1 competes with IRF4 binding at ISRE of the *Il9* locus to repress IL-9 expression. This is substantiated by (i) dose-dependent IRF1-mediated repression of IRF4-driven *Il9* promoter activity, (ii) dose-dependent suppression of IRF4-triggered IL-9 by overexpressed IRF1 in Th9 cells and (iii) decreased IRF4 binding in the presence of IRF1 occupancy at the *Il9* locus as shown by ChIP-Seq analyses. The previously defined IRF4-binding region in the *Il9* promoter harbours several ISRE (AANNGAAA) motifs^11^,^45^, which we analysed for IRF4-promoting function. We identified two functional IRF4-binding sites at −238/−226 bp from the transcription start site on the basis of *Il9* promoter activity assays performed with mutated ISRE. This was surprising because IRF4 peaks associated preferentially with AICE motifs on a genome-wide scale. As IRF4 (and IRF8), in comparison to other IRF family members including IRF1, displays lower affinity for binding to ISRE^34^, we presume those sites to be competitively bound by IRF1 leading to opposing regulation of *Il9* promoter activity in Th9 cells.

Transcription factor-induced transactivation is a multilevel event. After histone methylation-based initiation of active chromatin formation by pioneering factors, ensuing transcription factors such as EP300 enable the recruitment of transcriptional coactivator complexes, which then catalyse local histone acetylation (H3K27-Ac) required for gene transcription^49^. Both IRF1 and IRF4 have been shown to affect the histone acetylation status in immune cells^37^,^62^. We indeed detected opposite effects of IRF4 and IRF1 on the histone H4 acetylation status at *Il9* regulatory elements. Thus histone acetylation was decreased in IRF4-deficient Th9 cells as compared to WT Th9 cells, suggesting that IRF4 contributes to increased chromatin accessibility at the *Il9* locus. In contrast, IRF1 was crucial for IFN-γ-mediated suppression of permissive histone marks, implying that IRF1 limited the chromatin accessibility at the *Il9* CNS. Consistent with these counteracting activities, the recruitment of RNA pol II, which is crucial for transcriptional output, was decreased in IRF4-deficient Th9 cells, while it was increased under IRF1-deficiency as compared to WT cells. This points out that the changes in the acetylation status may contribute to the opposing regulation of IL-9 production by IRF4 and IRF1.

The model in which two members of one family competitively bind at the same cytokine locus has already been demonstrated for STAT3 and STAT5 in Th17 cells^63^. In this study, IL-2 signalling mediated its repressing effects via direct DNA binding of STAT5 at the *Il17* locus, which correlated with decreased STAT3 abundance as well as with decreased H3-Ac. This effect was mediated by increased binding of a histone deacetylator adaptor protein termed NCoR2. In our study, the general HDAC inhibitor trichostatin A was insufficient to oppose IFN-γ-mediated IL-9 repression, suggesting that the IFN-γ-IRF1 axis suppresses IL-9 production in an HDAC-independent manner. As IRF4 is known to recruit HAT p300 in Th0 and Th17 cells^30^, this could also apply for Th9 cells. Along with this idea, one could speculate that IRF1 counteracts the IRF4-mediated HAT recruitment or interferes with HAT function. However, more work has to be done to clarify this issue in detail. A recent study pointed to competitive binding of IRF1 and IRF4 at the *Il17* promoter thereby possibly regulating Th17/Tr1 commitment^61^. This is in agreement to our proposed model of IRF1/IRF4 competition as a mechanism to control Th differentiation.

Taken together, our study allows deeper insight into the function of IRF1 and IRF4 in Th9 cell differentiation and suggests for the first time that IRF1 competes with IRF4 to repress IL-9 production in Th9 cells. Furthermore, we demonstrate that IRF1 regulated gene expression positively on some, whereas negatively on other loci to shift Th9 cells towards a Th1-like phenotype. The knowledge of this IRF1-mediated transition of Th9 differentiation could prove useful for further unravelling Th9- and IFN-γ-associated immune responses during asthma as well as antitumour immunity. Altering the IRF1/IRF4 balance in the transcriptional network of Th9 cells to either increase or decrease Th9 responses might display a potential therapeutic approach.

## Methods

### Mice

C57Bl/6 mice were purchased from The Jackson Laboratory. *Irf1*^*−*/*−*^, *Irf4*^*−*/*−*^, *Rag2*^*−/−*^ and OTII mice expressing a transgenic TCR recognizing OVA_323-339_ were bred at the animal facility of the Biomedical Research Center, University of Marburg. *Irf1*^*−*/*−*^ mice were bred with OTII mice to produce *Irf1*^*−/−*^ OTII transgenic mice. *Stat1*^*−/−*^ mice were kindly provided by T. Decker (Max F. Perutz Laboratories, Department of Genetics, Microbiology and Immunobiology, University of Vienna, Austria). All mice used in the experiments were 8–12-week old, at C57Bl/6 background and sex- and age-matched. The experiments were approved by the local committee (Regierungspräsidium Gießen) and conducted according to the German animal protection law.

### Murine CD4^+^ T cell purification and *in vitro* differentiation

CD4^+^ T cells were purified by negative magnetic cell sorting (CD4^+^ T cell isolation kit, 130-104-454, Miltenyi) from the spleens and lymph nodes of 8–12-week-old mice. For some experiments, negatively purified CD4^+^ T cells were further sorted on a FACS AriaIII (BD Biosciences) to obtain naive CD44^*−*^CD62L^+^CD4^+^ T cells using anti-CD4-V450 (RM4-5, BD Biosciences), anti-CD44-PE (IM7, eBiosciences) and anti-CD62L-Alexa Fluor 700 (MEL-14, BD Pharmingen) monoclonal antibodies (mAbs) in a dilution of 1:500. Sorting purity was typically >97% in post-sort analysis. The cells were primed with plate-bound anti-CD3 mAb (3 μg ml^*−*1^, 145-2C11) and anti-CD28 mAb (5 μg ml^*−*1^, 37.51, both mAbs produced and purified ‘in house') in the presence of recombinant human (rh) IL-2 (50 U ml^*−*1^, Novartis) and anti-mouse-IFN-γ (5 μg ml^*−*1^, XMG1.2, produced and purified ‘in house'). The cells were cultured under: neutral (Th0) conditions, by the addition of anti-IL-4 (10% culture supernatant of clone 11B11), or Th9 conditions, by the addition of rhTGFβ (1.5 ng ml^*−*1^) and rmIL-4 (20 ng ml^*−*1^). Some cultures were supplemented with recombinant rat IFN-γ (5 ng ml^*−*1^, Peprotech) as indicated in the figure legends. Cells were usually harvested on day 2 for intracellular staining and qRT-PCR analysis. For kinetic experiments, the cells were analysed as indicated in figure legends.

For adoptive transfer experiments, CD4^+^ T cells were purified by negative magnetic cell sorting (CD4^+^ T cell isolation kit, 130-104-454, Miltenyi) from the spleens and lymph nodes of 8–12-week-old OTII and *Irf1*^*−/−*^OTII transgenic mice. The cells were differentiated under Th9 conditions in the presence of anti-mIFNγ (5 μg ml^*−*1^) and rat IFN-γ (5 ng ml^*−*1^). After 2 days, the cells were harvested, washed, counted and 5 × 10^5^ WT or *Irf1*^*−/−*^ OTII Th9 cells were transferred by intraperitonal (i.p.) injection into *Rag2*^*−/−*^ mice.

### Intracellular staining

For intracellular cytokine staining after differentiation, cells were restimulated with phorbol myristate acetate (50 ng ml^*−*1^) and ionomycin (1 μg ml^*−*1^) in the presence of brefeldin A (5 μg ml^*−*1^, both from Sigma) for 4 h, fixed with formaldehyde (2%) and stained with anti-IL-9-allophycocyanin (RM9A4, Biolegend) and anti-IFN-γ-phycoerythrin (PE) (XMG 1.2, eBioscience) mAbs. For intracellular staining of transcription factors after differentiation, cells were fixed with a Foxp3/Transcription Factor Fixation/Permeabilization Concentrate and Dilutent (eBioscience) and then stained with: anti-Foxp3-PE (FJK-16, eBioscience), anti-T-bet-PE (eBio4B10, eBioscience), anti-GATA-3-eF660 (TWAJ, eBioscience), anti-IRF4-AIFI647, (3E4, eBioscience) or anti-IRF1-PE (D5E4, Cell Signaling) mAbs. All antibodies were used in a 1:500 dilution. The cells were analysed by flow cytometry using a FACSCalibur or a FACSAriaIII (BD, Biosciences) with the DIVA software (BD, Biosciences) or the FlowJo Software (Tree Star).

### Phospho-flow

For staining of phosphorylated STAT1, human Th9 cells were harvested after 48 h of culture, rested for 4 h and treated with rhIFN-γ (100 ng ml^*−*1^) for 20 min. Then the cells were fixed and permeabilized according to BD Phosflow Protocol III using Lyse/Fix Buffer (557870, BD) and Perm Buffer III (558050, BD) and stained with anti-human phospho-STAT1 (Y701)-eFluor660 (KIKSI0803, eBioscience, 1:100).

### Stimulation and differentiation of human naive CD4^+^ T cells

PBMCs from healthy donors were labelled with anti-CD4-Pac Blue (RPA-T4, BioLegend), anti-CD45RA-FITC (HI100, eBioscience), anti-CD45RO-PE (UCHL1, BD) and anti-CD25-allophycocyanin (BC96, eBioscience) in 1:500 dilutions, and then naive CD4^+^ T cells (CD4^+^CD45RA^+^CD45RO^*−*^CD25^*−*^) were sorted using a FACS AriaIII (BD), the purity was typically >97% in post-sort analysis. Naive CD4^+^ T cells were cultured in RPMI complete media (5% human AB serum) with rhIL2 (30 U ml^*−*1^) and activated with Dynabeads Human T-Activator CD3/CD28 (111.31D, Invitrogen, T cell/bead=2:1). For induction of Th9 cell differentiation, rhIL-4 (30 ng ml^*−*1^) and rhTGF-β1 (5 ng ml^*−*1^) and either anti-hIFN-γ (5 μg ml^*−*1^) or rhIFN-γ (50 ng ml^*−*1^) were added to the cell cultures. Th0 cells were differentiated in the presence of anti-hIL-4 and anti-hIFN-γ (both 5 μg ml^*−*1^). Cells were analysed by flow cytometry for IRF1 and IRF4 presence after 24–36 h of culture and for IL-9 and IFN-γ production after 72 h of culture; supernatants were collected after 72 h and IL-9 was measured by enzyme-linked immunosorbent assay (ELISA; human IL-9 ELISA Max, 434705, BioLegend).

### RNA-mediated interference

An Amaxa Human T cell Nucleofector Kit (VPA 1002, Lonza) was used for knockdown of human IRF1 by siRNA in naive CD4^+^ T cells according to the manufacturer's instructions. Naive CD4^+^ T cells (5 × 10^6^) were activated with Dynabeads Human T-Activator CD3/CD28 overnight and then transduced with 3 μM IRF1-specific siRNA (ONTARGETplus, L011704-00) or control scrambled RNA (both Dharmacon) by the T-23 transfection program (Amaxa). Thereafter, the cells were cultured under Th9 conditions for additional 24–48 h, followed by flow cytometric analysis and evaluation of *IL9* mRNA by qRT-PCR and IL-9 production by ELISA.

### Immunoblot analysis

Purified murine CD4^+^ T cells were left unstimulated or stimulated as indicated in the figure legends. For detection of Tyr^694^STAT5 and Tyr^641^STAT6 phosphorylation, cells were preactivated under Th9 conditions in the presence of anti-mIFNγ (5 μg ml^*−*1^) and rIFN-γ (5 ng ml^*−*1^) for 2 days, then washed and rested in cytokine-free medium for 8 h. Preactivated cells were treated with rat IFN-γ (5 ng ml^*−*1^) in combination with either rmIL-4 (20 ng ml^*−*1^) or rhIL-2 (50 U ml^*−*1^) for 20 or 60 min. Whole-cell lysates were prepared as described previously^47^, 20 μg of total protein were loaded per lane and proteins were detected according to standard protocols. The following Abs were used: anti-β-actin (AC-15, Sigma-Aldrich), anti-IRF-1 (M-20, Santa Cruz), anti-IRF-4 (M-17, Santa Cruz), anti-STAT1 (#9172, Cell Signaling) anti-P-STAT1 (Tyr^701^, D4A7, Cell Signaling), anti-STAT5 (C-17, Santa Cruz), anti-P-STAT5 (Tyr^694^, C11C5, Cell Signaling), anti-STAT6 (M-20, Santa Cruz), anti-P-STAT6 (Tyr^641^, sc11762, Santa Cruz), anti-goat IgG-HRP (#sc-2020, Santa Cruz), anti-rabbit IgG-HRP (#sc-2004, Santa Cruz), and anti-mouse IgG-HRP (#sc-2055, Santa Cruz).

### Chromatin immunoprecipitation

Th9 cells cultured for 2 days in the presence of anti-mIFNγ (5 μg ml^*−*1^) and/or rat IFN-γ (5 ng ml^*−*1^) as indicated in the figure legends. In all, 2–5 × 10^6^ cells were crosslinked with 1% formaldehyde for 6 min at room temperature. Subsequently, ChIP was performed as previously described^48^. Lysed cells were sonicated in a Bioruptor Plus (Diagenode) with 30 s ON, 30 s OFF on high power output for 27–33 cycles at 4 °C. For immunoprecipitation, 2.5–4 μg of the following Abs were used: anti-IRF1 (M-20, sc-640x, Santa Cruz), anti-STAT1 (#9172, Cell Signaling), anti-RNA pol II (N-20, sc-899x, Santa Cruz), anti-H4-Ac (06-866, Millipore) and control IgG (#2729, Cell Signaling). qRT-PCR with the precipitated chromatin was performed to calculate the percentage of input. Primer sequences are provided in Supplementary Table 1. All amplifications were performed in triplicate with the QuantiTect SYBR Green PCR Kit (204143, Qiagen). Control ChIP was performed with a respective control antibody to ensure specificity. Values for nonspecific binding (as determined by control IgG) were subtracted. After normalization of the data according to the control, the specific pulldown (input %) was calculated.

### qRT-PCR

Total RNA was extracted from cells at day 1 or 2 of culture. For RNA isolation, the High Pure RNA Isolation Kit (11828665001, Roche) was used. Complementary DNA (cDNA) was synthesized with oligo(dT) primers using the RevertAid First Strand cDNA Synthesis Kit (K1621, Thermo Scientific) and gene expression was examined with an ABI Prism 7500 Sequence Detection System (Applied Biosystems) using the SYBR green I qPCR Core Kit (10-SN10-05NR, Eurogentec). Levels of each gene were normalized to hypoxanthine-guanine phosphoribosyl transferase (*Hprt1*) expression using the ΔΔCt method, with the lowest experimental value set to 1. The primer sets are provided in Supplementary Table 1.

### Luciferase reporter assay

Naive CD62L^**+**^CD4^+^ T cells from C57Bl/6 mice were isolated and stimulated for 2 days under Th0 conditions. The preactivated Th0 cells were transfected with the Mouse T Cell Transfection Kit (Amaxa) according to the manufacturer's instructions with 0.5 μg of reporter vector coding for the Firefly Luciferase (pGL3 basic, Promega) under the control of the *Il9* promoter (CNS1) (−610 to +32)^11^ or mutants within *Il9* promoter-IRF-III (M1 or M3). Additionally, cells were transfected with 2 μg of the IRF4^11^ in pcDNA 3.1(+) (Invitrogen) vector and/or with 2 μg of the IRF1 or IRF1ΔDBD, all in pmax (Lonza) vectors and/or with 2 μg of respective control vectors. To control transfection efficiency and absolute cell numbers, cells were co-transfected with 0.5 μg of the pRSV β-gal plasmid. In experiments with IRF4- and IRF1-expressing vectors, Th0 cells were transfected with the total amount of 5 μg DNA. Twenty-four hours after transfection, cells were stimulated with phorbol myristate acetate (20 ng ml^*−*1^) and ionomycin (370 ng ml^*−*1^) for further 24 h. Harvested cells were washed and lysed and the luciferase activity was measured for each sample and divided by the β-galactosidase activity of the sample to obtain relative luciferase activity (relative light units).

For the generation of the IRF1 vector, the gene encoding murine full-length IRF1 (accession number NM_008390) was amplified by PCR with the following primers: forward: 5′-ATG CCA ATC ACT CGA ATG-3′; and reverse 5′-CAG AGA CCC AAA CTA TGG TG-3′. Mutated IRF1 lacking the DNA-binding domain (IRF1ΔDBD) was amplified with the following primers: forward: 5′-ATG CTC ACC AGG AAC CAG AGG-3′, and reverse 5′-CAG AGA CCC AAA CTA TGG TG-3′. The PCR products were control digested with SalI and Kpn1 and cloned into the vector pmax (Lonza). Constructs were sequenced to prove authenticity.

Mutations in potential IRF-binding sites within the *Il9* CNS1-IRF-III site were generated using the QuickChange Site-Directed Mutagenesis Kit (Agilent Laboratories) and the following primers: M1.for: 5′-CTG AAA TAC TAA AGG AGG AGT TAA AGA TCT AGC CCC AAC CCC CTT-3′, M1.rev: 5′-AAG GGG GTT GGG GCT AGA TCT TTA ACT CCT CCT TTA GTA TTT CAG-3′, M3.for: 5′-CTT CAA ATA GTC GGG TTC TGA GGT ACT AAA GGA AAA GTT AAA GAT CTA GCC C-3′, and M3.rev: 5′-GGG CTA GAT CTT TAA CTT TTC CTT TAG TAC CTC AGA ACC CGA CTA TTT GAA G-3′. Constructs were verified by DNA sequencing.

### Retroviral transduction

The gene encoding murine full-length IRF1 (accession number NM_008390) was amplified by PCR with the following primers: forward: 5′-CCA TGC CAA TCA CTC GAA TG-3′; and reverse 5′-CAG AGA CCC AAA CTA TGG TG-3′. The PCR product was control digested with BglII and EcoRI and cloned into the retroviral vector pMSCV containing the IRES-regulated gene for GFP or Thy.1. The construct was sequenced to prove authenticity. The retroviral vector pMSCV containing IRF4-IRES-GFP, the empty control vector containing IRES-GFP and transduction were described previously^64^ WT, *Irf4*^*−/−*^ or *Irf1*^*−/−*^ CD4^+^ T cells were primed under Th0 conditions and, on day +1, were infected with an IRF1-expressing retrovirus (IRF1-RV), IRF1 mutant-expressing retrovirus (IRF1ΔDBD-RV), IRF4-expressing retrovirus (IRF4-RV) or a control retrovirus (Control-RV). Transfected cells were cultured under Th9 conditions (TGF-β and IL-4) for 48 h, then washed and rested in media containing rhIL-2 (50 U) and αmIFN-γ for 72 h. Thereafter, the cells were re-cultured under Th9 conditions for additional 48 h, then washed, restimulated and analysed by flow cytometry for intracellular IL-9, or without restimulation, the cells were analysed for intracellular IRF1 and IRF4 levels.

### Asthma adoptive transfer model

*Rag2*^*−/−*^ mice received 5 × 10^5^ WT or *Irf1*^*−/−*^ OTII-Th9 cells on day 0 by i.p. injection. Mice were then challenged via the airways with nebulized OVA (1% in saline) with an ultrasonic nebulizer (NE-U17; Omron, Hoofdrop, The Netherlands) for 20 min daily from day 1 to day 6. To neutralize IL-9 cytokine *in vivo*, mice were injected i.p. either with 30 μg anti-IL-9 (MM9C1, produced and purified ‘in house') or rat IgG (Sigma) antibody on days +1, +3 and +5. On day 7, BAL and lung tissue were collected.

### BAL and lung single-cell preparation

Cells were isolated by lavage of the lungs via a tracheal tube with PBS (1 ml). Numbers of BAL cells were counted with trypan blue dye exclusion. Differential cell counts were made from cytocentrifuged preparations, fixed and stained with the Microscopy hemacolor set (Merck, Darmstadt, Germany). The percentage and absolute numbers of each cell type were calculated.

Lungs were fixed by inflation (1 ml) and immersion in 10% formalin and embedded in paraffin. Tissue sections were stained with haematoxylin and eosin and periodic acid-Schiff. Slides were examined in a blinded manner by two experienced observers by microscopy (BX40, Olympus, Germany) and peribronchial and perivascular inflammation was graded by a semiquantitative score (no inflammation=0, severe inflammation=10). For each slide, five randomly chosen areas were scored. As for periodic acid-Schiff-stained slides, the number of goblet cells was analysed by the imaging software (Analysis, Soft Imaging Systems, Stuttgart, Germany). The number of mucus-containing cells per millimetre of basement membrane was determined.

Lungs were minced and enzymatically digested for 1 h with collagenase D (1 mg ml^*−*1^) and DNase I (20 μg ml^*−*1^, both from Roche) in RPMI medium. Dispersed cells were passed through a 30-μm cell strainer (Miltenyi Biotec). Isolated lung cells (4 × 10^5^ in 200 μl) were stimulated with 2 mM OVA_323–339_ for 72 h, and the supernatants were analysed for the amounts of IL-9, IFN-γ and IL-13 by ELISA (IL-13 and IFN-γ, from BD Pharmigen, for murine IL-9 detection, mAbs 229.4 and C12 were used, produced and purified ‘in house').

### RNA-Seq and bioinformatical analysis

Naive CD4^+^ T cells isolated from WT and *Irf1*^*−/−*^ mice were cultured for 2 days under Th9 conditions in the presence of IFN-γ (5 ng ml^*−*1^). RNA was purified with the RNeasy Plus Mini Kit according to the manufacturer's protocol (Qiagen). RNA was quantified with a Qubit 2.0 fluorometer (Invitrogen) and the quality was assessed on a Bioanalyzer 2100 (Agilent) using a RNA 6000 Nano chip (Agilent). Samples with an RNA integrity number of >8 were used for library preparation. Barcoded mRNA-seq cDNA libraries were prepared from 400 ng of total RNA using NEBNext Poly(A) mRNA Magnetic Isolation Module and NEBNext Ultra RNA Library Prep Kit for Illumina according to the manual. Quantity was assessed using Invitrogen's Qubit HS Assay Kit and library size was determined using Agilent's 2100 Bioanalyzer HS DNA assay. Barcoded RNA-Seq libraries were onboard clustered using HiSeq Rapid SR Cluster Kit v2 using 8 pM and 50 bps were sequenced on the Illumina HiSeq2500 using the HiSeq Rapid SBS Kit v2 (50 Cycle). The raw output data of the HiSeq was preprocessed according to the Illumina standard protocol. Quality control on the sequencing data was performed with the FastQC tool (available at http://www.bioinformatics.babraham.ac.uk/projects/fastqc/), as well as the comprehensive Qorts suite^65^. Inspecting the produced reports, all samples were deemed of good quality for further processing. Short reads alignment was performed with the ENSEMBL Mus_musculus.GRCm38 chosen as the reference genome. The corresponding annotation (ENSEMBL v76) was also retrieved from the ENSEMBL FTP website (http://www.ensembl.org/info/data/ftp/index.html). The STAR aligner (version 2.4.0b) was used to perform mapping to the reference genome. Alignments were processed with the featureCounts^66^ function of the Rsubread package, using the annotation file, also used for supporting the alignment. Exploratory data analysis was performed with the pcaExplorer package. Differential expression analysis was performed with DESeq2 (version 1.12.3, https://www.ncbi.nlm.nih.gov/pubmed/25516281), setting the False Discovery Rate to 0.05 (ref. 67). Gene expression profiles were plotted as heatmaps (colour-coded *z*-scores for each fragments per kilobase of exon per million fragments mapped value) with the R statistical software. GSEA was performed as previously described^68^.

### ChIP-Seq and bioinformatics analysis

Naive CD4^+^ T cells were cultured under Th9 conditions for 2 days, then rested for 3 days and restimulated under Th9 conditions with or without IFN-γ treatment for additional 2 days. ChIP-Seq was performed as previously described^47^. Shortly, for IRF1 and IRF4 ChIP-seq, 10^7^ cells were crosslinked with 1% paraformaldehyde for 30 min, and for H3K27ac ChIP-seq, 3 × 10^5^ cells were crosslinked with 1% paraformaldehyde for 5 min. Subsequently, cells were lysed and sonicated using Digital Sonifier (Branson). For immunoprecipitation, the lysate was incubated overnight with 50 μl DynaBeads IgG magnetic beads (Thermo Fisher) that had been preincubated with 2.5 μg of the following antibodies: anti-IRF1 (abcam, ab186384), anti-IRF1 (M-20, sc-640x, Santa Cruz), anti-IRF4 (abcam, ab101168), and anti-H3K27ac (GeneTex, GEX60815). After washing, elution and reverse crosslinking at 65 °C overnight, precipitated DNA was purified using the MinElute PCR Purification Kit (Qiagen). For IRF1 and IRF4 CHIP-seq, purified DNA was fragmented using Covaris Focused-ultrasonicator S220 (Covaris) before library preparation. Library was constructed using the KAPA Library Preparation Kit Ion Torrent (KAPA Biosystems) according to the manufacturer's instructions and sequenced using Ion S5 (Thermo Fisher). Quality control on the raw data was performed with FastQC. Mapping was performed against the Mus Musculus GRCm38 reference with bowtie2, version 2.2.9. The aligned files were converted to bam, sorted and indexed for the subsequent steps. Additional controls to check antibody enrichment was done with the NGSplot tool and inspecting the metagene profiles combined with basic mapping statistics. Peak calling was performed with MACS2, using the sharp peak calling algorithm. Downstream analyses were performed with the ChIPpeakAnno package. Promoter regions were defined as the genomic intervals spanning 500 bp upstream and 500 bp downstream annotated Transcription Start Sites (retrieved from the ENSEMBL annotation). Occupancy levels were quantified with the summarizeOverlaps function of the GenomicAlignments package, and values were subsequently normalized and log2 transformed (after adding1 as pseudocount). Functional annotation of the affected promoters was performed with topGO. Enriched GO Terms in the genes annotated to Irf1 peaks was performed with the topGO Bioconductor package, using the elim algorithm. Motif analysis was performed with the Biostrings package, and their occurrencies are presented with the functions in the UpSetR package (EICE: GGANTGA, AICE: TGAG/CTCA, ISRE AANNGAAA). The MEME/DREME software tool^69^ to search for over-represented motifs was used.

### Statistical analysis

For statistical analysis, a two-tailed Student's *t*-test was performed using the GraphPad Prism software (GraphPad Software). For analysis of data obtained in the allergic airway adoptive transfer model, differences between groups were evaluated by one-way ANOVA test with Tukey's post-test. *P* values are indicated as follows: *P<0.05; **P<0.005; ***P<0.001.

### Data availability

Sequence data that support the findings of this study have been deposited in GEO with the primary accession codes GSE96818 and GSE96699. The authors declare that all other data supporting the findings of this study are available within the article and its Supplementary Information files.

## Additional information

**How to cite this article:** Campos Carrascosa, L. *et al*. Reciprocal regulation of the *Il9* locus by counteracting activities of transcription factors IRF1 and IRF4. *Nat. Commun.* **8,** 15366 doi: 10.1038/ncomms15366 (2017).

**Publisher's note:** Springer Nature remains neutral with regard to jurisdictional claims in published maps and institutional affiliations.

## Supplementary Material

Supplementary Information Supplementary Figures and Supplementary Table

Supplementary Data 1 GO enrichment analysis of IRF1 targets.
