# Supplementary material for: Reciprocal regulation of the Il9 locus by counteracting activities of transcription factors IRF1 and IRF4
Source: Nat Commun. 2017 May 12;8:15366. doi: 10.1038/ncomms15366 (PMC5437292; doi:10.1038/ncomms15366)
Supplement: Supplementary Information — Supplementary Figures and Supplementary Table [file ncomms15366-s1.pdf]

Supplementary Figure 1

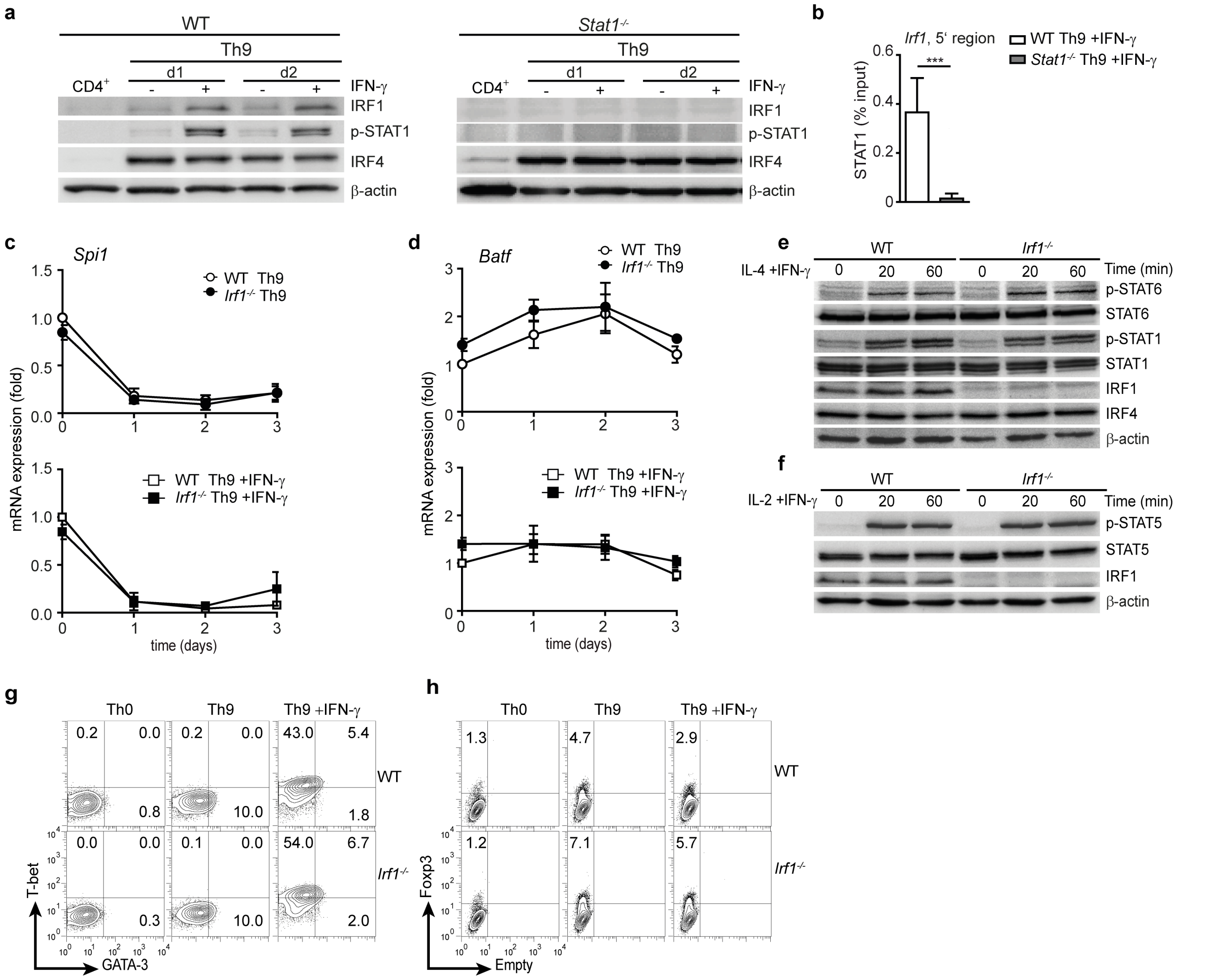

Supplementary Figure 1. IFN-γ/STAT1-induced IRF1 does not affect expression of Th9-related transcription factors. Purified $CD4^+$ T cells (**a,b,e,f**) or sorted naïve $CD44^-CD62L^+CD4^+$ T cells (**c,d,g,h**) were isolated from WT, *Irf1*$^{-/-}$ or *Stat1*$^{-/-}$ mice and then treated under Th9 (TGF-β+IL-4) or Th0 (without skewing cytokines) conditions with/without IFN-γ as indicated. (**a**) Immunoblot analysis of indicated proteins in $CD4^+$ T cells untreated or stimulated as indicated for one or two days (d1, d2). (**b**) ChIP analysis of STAT1 occupancy at the *Irf1* 5' proximal promoter in WT or *Stat1*$^{-/-}$ T cells stimulated for 13 h under Th9 conditions in the presence orf absence of IFN-γ (n=5, mean ±SD of percent input with substraction of control IgG). Data are combined from two independent experiments. (**c,d**) Kinetic expression of *Spi1* (**c**) and *Batf* (**d**) mRNA (qRT-PCR). mRNA expresion was normalized to *Hprt1* and relative expression was calculated by setting of the value for WT naive $CD4^+$ T cells at the time point 0 to 1. Data represent three individual experiments combined (mean ±SD). (**e,f**) Immunoblot analysis for the indicated proteins in Th9 cells, harvested after two days, rested in cytokine-free medium for 8 h and then treated for indicated time periods with IFN-γ in combination with rmIL-4 (**e**) or with rhIL-2 (**f**). (**g,h**) Flow cytometric analysis of cells intracellularly stained for T-bet and GATA3 (**g**) or Foxp3 (**h**) on day two of culture. (**a,e-h**) Results are representative of three independent experiments. ***$p<0.001$, 2-tailed Student's t test

Supplementary Figure 2

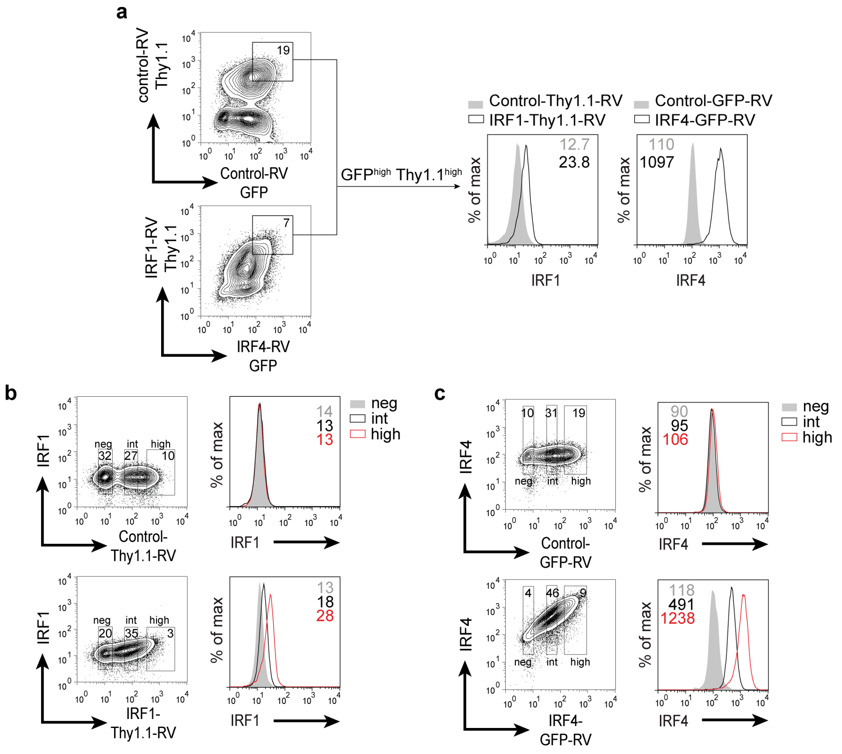

Supplementary Figure 2. The relative intensity of GFP- and Thy1.1-tags represents the relative expression of IRF4 and IRF1 in *Irf4*$^{-/-}$ Th9 cells upon IRF4-GFP-RV and IRF1-Thy1.1-RV double overexpression. *Irf4*$^{-/-}$ CD4^{+} T cells were activated under Th0 condition overnight and spin-infected with the following retroviruses as indicated: control-GFP-RV, IRF4-GFP-RV, control-Thy1.1-RV and IRF1-Thy1.1-RV. Thereafter, cells were cultured under Th9 conditions for two further days, rested for three days and restimulated under Th9 conditions for additional two days. (**a**) Highly double positive cells (GFP^{hi}Thy1.1^{hi}) were selected for further analysis of IRF1 and IRF4 expression. (**b,c**) Three subsets (neg, int, high) of cells expressing increasing levels of GFP and Thy1.1 were selected for analysis of IRF1 (**b**) and IRF4 (**c**) expression by each subset. (**a-c**) Numbers in gates represent % of gated cells, whereas numbers in histograms give the mean flurescence intensity (MFI). Data are representative of three independent experiments.

Supplementary Figure 3

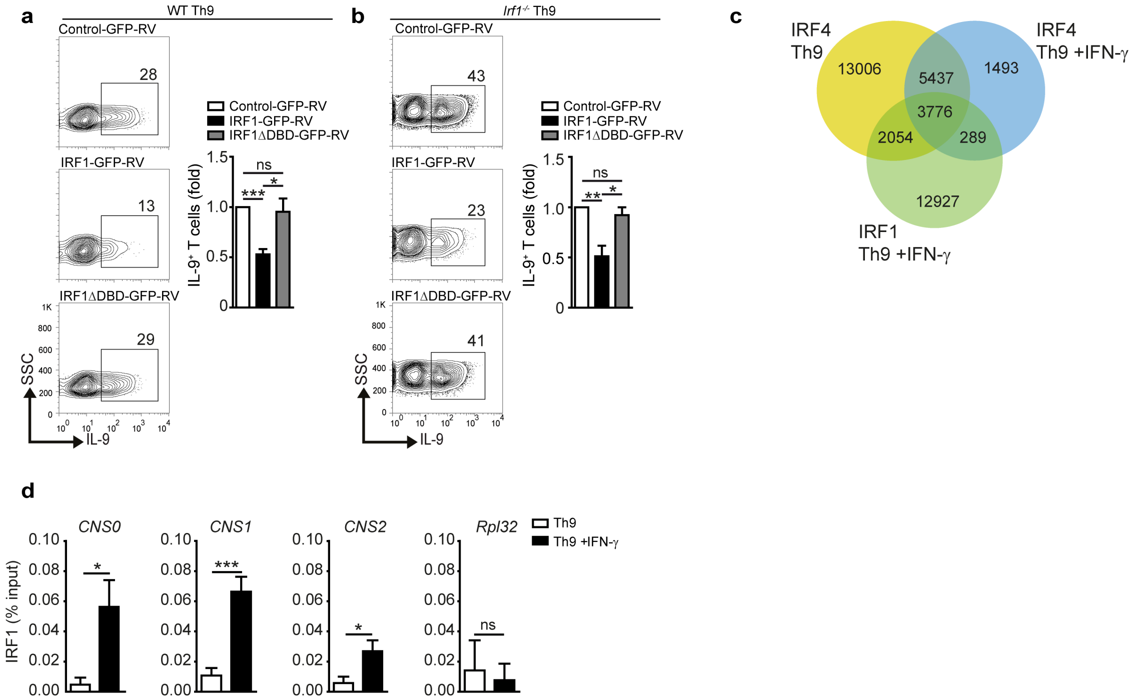

Supplementary Figure 3. IRF1 directly binds to the *Il9* locus and DBD-dependently suppresses IL-9 production in Th9 cells. (**a**) WT or (**b**) *Irf1-/-* CD4+ T cells were cultured under Th0 condition over night and then spin-infected with the following retroviruses as indicated: control-GFP-RV, IRF1-GFP-RV, IRF1ΔDBD. Thereafter, cells were cultured under Th9 conditions for further two days, rested for three days and restimulated under Th9 conditions for additional two days. Highly GFP-expressing cells were selected for further analysis of IL-9 production. Contour-plots show one representative of three independent experiments. Bars to the right give three independent experiments combined and give mean ±SD. (**c**) Venn diagramm of IRF1 and IRF4 peaks in Th9 cells in absence or presence of IFN-γ as indicated. (**d**) ChIP analysis of IRF1 occupancy at the indicated loci (CNS0,1,2 of the *Il9* locus) in WT CD4+ T cells stimulated for 13 h under Th9 conditions with or without IFN-γ (three independent experiments combined, mean ±SD of percent input with substraction of control IgG). *p<0.05, **p<0.005, ***p<0.001 (2-tailed Student´s t test).

Supplementary Figure 4

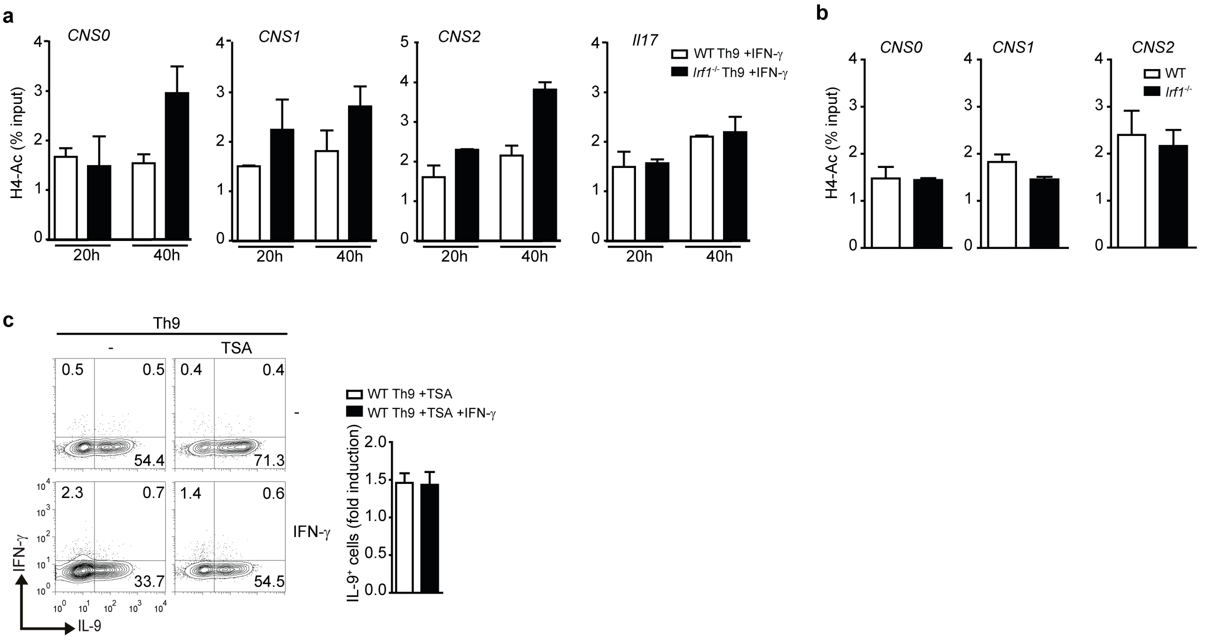

Supplementary Figure 4. IRF1 suppresses the acetylation of histone H4 at *Il9* CNSs independently of HDAC recruitment. (**a,b**) ChIP assays of acetylated (Ac) histone H4 at CNSs of the *Il9* gene. (**a**) WT and *Irf1*$^{-/-}$ CD4^{+} T cells were (**a**) cultured for 20 or 40 h under Th9 conditions with or without IFN-γ or (**b**) were directly analyzed. (**a,b**) The same chromatin was used for control ChIP experiments with control IgG. Precipitated DNA is presented relative to input (% input). Values for nonspecific binding (as determined by using control IgG) were subtracted. (**c**) WT CD4^{+} T cells were cultured under Th9 condition with/without IFN-γ or TSA (1 nM) for two days and then analyzed for IL-9 and IFN-γ levels by flow cytometry. Bars to the right show fold induction of IL-9^{+} T cells relative to non-TSA-treated cells and are combined from three independent experiments, mean ±SD. (**a-c**) Data and contour-plot are representative for three independent experiments.

Supplementary Figure 5

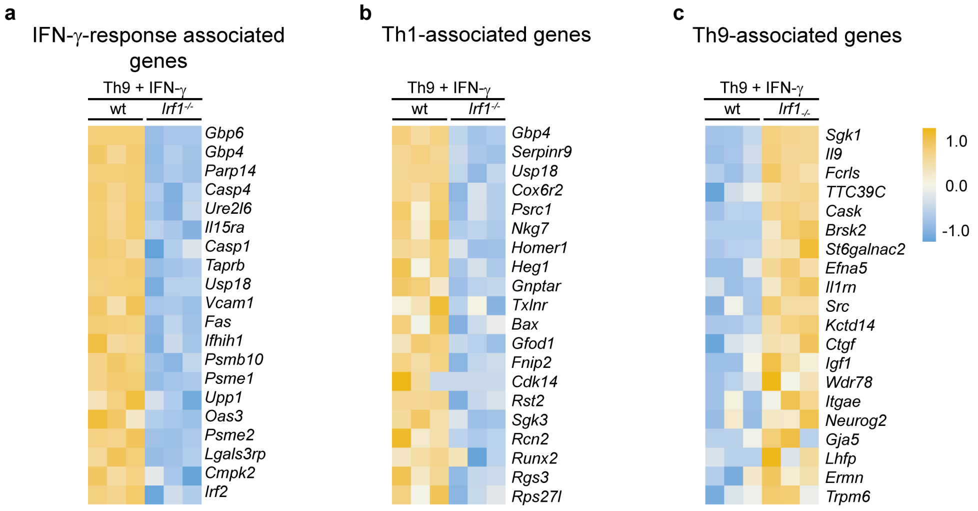

Supplementary Figure 5. IRF1 modulates the fate of Th9 cells towards IFN/Th1 gene signature. Naïve CD4+ T cells isolated from WT and *Irf1-/-* mice were cultured under Th9 conditions in the presence of IFN-γ. Total RNA was purified from the cells and RNA-Seq was performed from three independent biological samples. Heatmap is color-coded by z-score. Displayed are top 20 core enriched hits of each GSEA of (**a**) 'Hallmark IFN-γ response', (**b**) 'Th1-associated' or (**c**) 'Th9-associated genes'.

Supplementary Figure 6

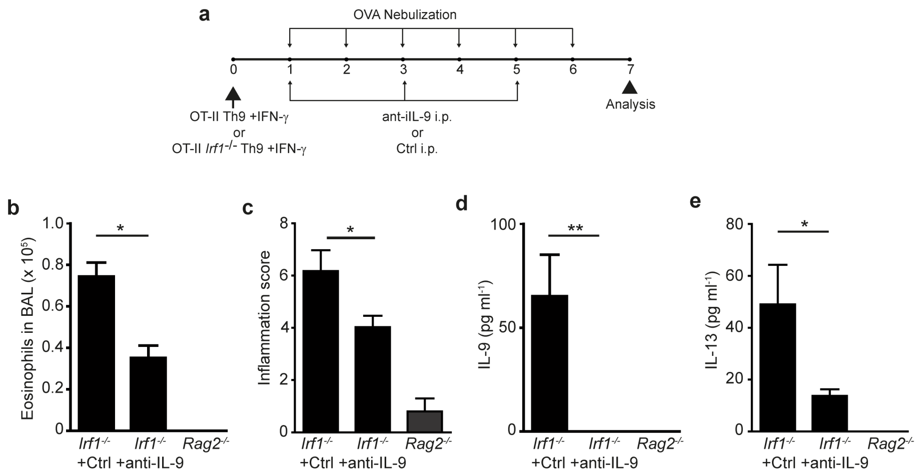

Supplementary Figure 6. The IFN-γ/IRF1 pathway restricts allergic airway inflammation IL-9-dependently. (**a**) Purified CD4$^+$ T cells from WT and *Irf1*$^{-/-}$ OTII mice cells were polarized under Th9 conditions with IFN-γ for two days, then transferred into *Rag2*$^{-/-}$ mice, which were thereafter challenged with nebulized OVA for six days. Some of the recipient mice were treated with IL-9 neutralizing antibodies (anti-IL-9) or control rat IgG (Ctrl) antibodies on the indicated days. Recipient mice were sacrificed 24 h after the last challenge. (**b**) Cell numbers in the BAL. (**c**) Tissue inflammation was evaluated with hematoxylin and eosin (H&E) staining. Slides were scored for peribronchial and perivascular inflammation with semiquantitative score from 0 to 10. (**d,e**) Lung cells were stimulated with 2mM $OVA_{323-339}$ for three days. IL-9 and IL-13 production was determined in supernatants by ELISA. (**b**-**e**) Data from one representative experiment (n=5 mice per group). The experiments were repeated four times with consistent results. *p<0.05, **p<0.005 by one-way ANOVA with Tukey´s post-test (**b,c**) or 1-tailed Student's *t* test (**d,e**).

Supplementary Table 1. Primers for ChIP and qPCR analyses

ChIP primers	Sequences	Location (to TSS)
Il9 CNS0	5’-ATGCGGAATGGGTTTTCACT-3’<br>5’-AAGCTCCACACACTTAGTTTGT-3’	-6287 to -6093
Il9 CNS1	5’-CCCTGTAACTCACTGTCTATCAGC-3’<br>5’-GCAGGAATTCTGGTTGTGAG-3’	-375 to -270
Il9 CNS2	5’-TCACCCACTTTAGTCCTTTCAAAA-3’<br>5’-AATTACAGAATTTTGCCCCAGGTCCTG-3’	+4888 to +4983
Il9 gene	5’-TGATTGTACCACACCGTGCT-3’<br>5’-TATCCTTTTCACCCGATGGA-3’	+1557 to +1657
Il17 promoter	5’-GCTCTCCCTGGACTCATGTT-3’<br>5’-TGGTTCTGTGCTGACCTCAT-3’	-131 to +74
RpL32 promoter	5’-TCATTTCTCAGGCACATCTT-3’<br>5’-ACTCACCGTAAAACAGATGG-3’	-116 to +56
Irf1 promoter	5’-TACAACAGCCTGATTTCCCC-3’<br>5’-TACCTCGACGAAGGAGTGGT-3’	-104 to +64

qRT-PCR primers	Sequences
Spi1	5’-AAC AGA TGC ACG TCC TCG AT-3’<br>5’-GGG CTG GGG ACA AGG TTT GAT AAG-3’
Batf	5’-GAAGAATCGCATCGCTGC-3’<br>5’-CGTTCTGTTTCTCCAGGT-3’
Hprt1	5’-CTGGTGAAAAGGACCTCTCG-3’<br>5’-TGAAGTACTCATTATAGTCAAGGGCA-3’
IL9	5’-CATGCAAACAAGATACCCACTG-3’<br>5’-TTGCCTCTCATCCCTCTCATC-3’
IFNG	5’-TGG GTT CTC TTG GCT GTT ACT G-3’<br>5’-ACA CTC TTT TGG ATG CTC TGG TC-3’
IRF1	5’-TGGCTGGGACATCAACAAGG-3’<br>5’-CTGCCCTTGTTCCTGCTCTG-3’
IRF4	5’-CTCTTTGACACACAGCAGTTCTTG-3’<br>5’-TTCTGGTAAATCGTAGCCCCTC-3’

18S	5'-AGTCCCTGCCCTTTGTACACA-3'<br>5'-GATCCGAGGGCCTCACTAAAC-3'
